# Free Fatty Acids and Their Inflammatory Derivatives Affect BDNF in Stroke Patients

**DOI:** 10.1155/2020/6676247

**Published:** 2020-12-03

**Authors:** Dariusz Kotlega, Agnieszka Zembron-Lacny, Barbara Morawin, Monika Golab-Janowska, Przemyslaw Nowacki, Malgorzata Szczuko

**Affiliations:** ^1^Department of Neurology, Pomeranian Medical University in Szczecin, Poland; ^2^Department of Applied and Clinical Physiology, Collegium Medicum, University of Zielona Gora, Poland; ^3^Department of Human Nutrition and Metabolomics, Pomeranian Medical University in Szczecin, Poland

## Abstract

**Objective:**

The neurotrophin brain-derived neurotrophic factor (BDNF) affects poststroke functional outcome, neurogenesis, neuroprotection, and neuroplasticity. Its level is related to the diet and nutritional status, and more specifically, it is free fatty acids (FFAs) and eicosanoids that can have an impact on the BDNF level. The aim of this study was to analyze the potential impact of FFAs and eicosanoids on the BDNF level in stroke patients. *Material and Methods*. Seventy-three ischemic stroke patients were prospectively enrolled in the study. Laboratory tests were performed in all subjects, including the levels of FFAs, eicosanoids, and BDNF. FFAs and inflammatory metabolites were determined by gas chromatography and liquid chromatography, while BDNF was evaluated by the immune-enzymatic method (ELISA).

**Results:**

The plasma level of BDNF negatively correlated with C22:1n9 13 erucic acid, C18:3n3 linolenic acid (ALA), and lipoxin A4 15-epi-LxA4. A direct association was observed in relation to BDNF and C16:1 palmitoleic acid and C20:3n6 eicosatrienoic acid (dihomo-gamma-linolenic acid (DGLA)).

**Conclusions:**

Saturated fatty acids and omega-3 and omega-9 erucic acids can affect signaling in the BDNF synthesis resulting in the decrease in BDNF. There is a beneficial effect of DGLA on the BDNF level, while the effect of ALA on BDNF can be inhibitory. Specialized proresolving lipid mediators can play a role in the BDNF metabolism. BDNF can interact with inflammation as the risk factor in the cardiovascular disorders, including stroke.

## 1. Introduction

### 1.1. The Role of Brain-Derived Neurotrophic Factor

The brain-derived neurotrophic factor (BDNF) can serve as a main regulator of plasticity in an activity-dependent manner in the central nervous system [1]. Nerve growth factor (NGF), BDNF, neurotrophin-3 (NT-3), and neurotrophin-4/5 (NT-4/5) regulate the neuronal architecture and belong to the family of neurotrophins. The receptors of tropomyosin receptor kinase (Trk) mediate the impact of neurotrophins on signaling pathways. This leads to the activation of survival, differentiation, and growth [2]. The memory and learning processes are connected with the modulation of the synaptic excitatory strength that can be regulated by neurotrophins. They also regulate hippocampal long-term potentiation [1]. BDNF is expressed in hippocampal neurons to a large extent, and it binds to the tropomyosin receptor kinase B (TrkB), regulates synaptic plasticity, and supports regulation of neuronal morphology and circuits involved in the processes of memory and learning. Diverse mechanisms are involved in the regulation of its activity [3]. Poststroke rehabilitation involves motor and communication learning. The functions of BDNF, including neurogenesis, neuroprotection, and neuroplasticity, can give a chance of developing and improving rehabilitation options after stroke. The refinement of neuroplasticity can be supported by this neurotrophin with the facilitation of long-term activation, remodeling, and strengthening of the neuronal connections and dendritic growth [4]. BDNF promotes myelination, differentiation, and proliferation of oligodendroglia through the oligodendrocyte precursor cells (OPC) [5].

Clinical studies indicate the role of BDNF as a risk factor for stroke and a marker of the prognosis, mortality, and functional outcome in stroke survivors [6]. According to the Danish National Registry, low plasma levels of BDNF in elderly women are connected with greater mortality, regardless of other factors [7]. The level of BDNF can be increased in stroke patients after atorvastatin treatment. A beneficial effect is also observed in relation to neurological outcomes measured with the use of the modified Rankin Scale (mRS) and Barthel Index (BI) [8].

### 1.2. Association between BDNF and Nutrition

The *α*-linolenic acid (ALA; 18:3n-3) is a polyunsaturated omega-3 fatty acid, and its supplementation for one week in healthy subjects was found to lead to an increase in the BDNF level [9]. The n-3 polyunsaturated fatty acid (PUFA) intake was assessed with the use of a questionnaire and indicated a direct effect on the BDNF level [10]. In the experimental model of stroke, the pretreatment with flaxseed oil that contains mainly ALA and linoleic acid (n-6 PUFA) increased the level of BDNF in the cortex [11]. The experimental models showed that docosahexaenoic acid (DHA) supplementation directly correlated with the BDNF level in traumatic brain injury, depression, and age-related decline in cognitive function [12]. In healthy subjects, raising the level of FFAs resulted in a significant decrease by 43% in serum BDNF after 360 min. The plasma BDNF level decreased by 27.8% after a high-fat meal [13]. A high-fat diet was shown to decrease neural progenitor cell proliferation and neurogenesis, to increase lipid peroxidation, and to decrease BDNF in the hippocampus, whereas BDNF had a protective effect on neural progenitor cells against lipid peroxidation [14]. On the other hand, a dietary quality assessed with the use of the Australian Dietary Quality Score did not affect the level of BDNF in depression sufferers [15]. The n-3 PUFA supplementation for 6 months in patients after the first episode of schizophrenia was associated with an increased level of BDNF, but no effect on BDNF was recorded in patients with posttraumatic stress disorder or depression [16–18]. Interestingly, the level of BDNF was associated positively with cardiovascular risk factors such as diastolic blood pressure, BMI, LDL cholesterol, total cholesterol, and triglycerides [19]. Another study showed that weight loss interventions decreased the level of BDNF in a manner dependent on methods (diet and/or exercise) and sex [20]. Contrastingly, some observations showed that BDNF inversely correlated with the percentage of body fat, fasting glucose, triglycerides, and insulin sensitivity [21]. Increased levels of oxLDL and hsCRP and the higher TC/HDL ratio (atherogenic index) were connected with lower levels of BDNF. On the other hand, better results obtained in the ergometer test were associated with increased levels of plasma BDNF [22]. Moreover, type 2 diabetes patients have lower plasma BDNF levels compared to healthy individuals [23]. Dyslipidemia and hypertension are major risk factors for coronary heart disease (CHD), and BDNF can play a regulatory role in blood pressure and lipid metabolism. However, it is not clear whether these associations are causal or the disrupted metabolism of lipids and hypertension provoke compensatory changes in serum BDNF levels. The inflammatory process can be a link between BDNF and dyslipidemia [24].

The free fatty acids and diet can be responsible for a potential link between cardiovascular risk factors and inflammation. The FFAs have an impact on the synthesis of inflammatory molecules and modulate neurotransmission and receptor function [25]. The n-3 PUFA have anti-inflammatory properties and are used in the synthesis of eicosanoids, while polyunsaturated fatty acids are used in the synthesis of leukotrienes and prostaglandins [26]. Protectins, resolvins, and maresins are anti-inflammatory specialized proresolving mediators (SPMs) that are synthesized from n-3 PUFA (DHA) [27, 28]. Other representatives of SPMs include lipoxins that are metabolized from arachidonic acid (AA) [29]. The level of FFAs as determined by a diet has an impact on inflammatory molecules such as CRP, E-selectin, and Il-6 [30, 31]. Lower levels of inflammatory mediators are observed in diets rich in n-3 PUFA and in the Mediterranean diet [32, 33]. The BDNF-induced activation of its receptor—tropomyosin receptor kinase B—in the cerebral arteries promotes the synthesis of prostacyclin [34]. Such associations indicate the possibility that BDNF affects inflammatory metabolites or it can be influenced by FFAs and eicosanoids.

### 1.3. Association between BDNF and Eicosanoids

The intake and the level of FFAs have an impact on the eicosanoids that are their inflammatory metabolites. The BDNF synthesis is induced by prostaglandins, especially PGE2 and PGD2 [35]. There is a potential link between eicosanoids including SPMs and BDNF. Resolvins and lipoxins activate diverse cells and stimulate endothelial synthesis of vasoprotective prostacyclin (PGI2) and nitric oxide (NO). These eicosanoids also stimulate macrophage phagocytosis through G protein-coupled receptors (GPCRs) leading to decreased synthesis of proinflammatory cytokines from dendritic cells, neutrophils, macrophages, and endothelial cells. On the other hand, the synthesis of anti-inflammatory cytokines is increased [36]. BDNF activates signaling through the TrkB receptor and activates the biosynthesis of prostacyclin in the cerebral arteries [34].

The aim of the study was to evaluate the impact of FFAs and their inflammatory metabolites on the level of BDNF in stroke patients. This is the first study to have analyzed these potential associations.

## 2. Material and Methods

The protocol of the study was approved by the Ethics Committee in Zielona Góra (decision number 08/73/2017, Feb 27, 2017). The study was conducted in accordance with the Declaration of Helsinki. All patients signed a written informed consent form to participate in the study.

### 2.1. Subjects

A prospective study included seventy-three patients with the diagnosis of ischemic stroke. All participants were recruited during the hospital stay in the department of neurology in the district hospital in Poland. Cerebral ischemic stroke was the main inclusion criterion. The diagnosis was established on the basis of the symptoms and brain imaging (MRI or CT). Routine laboratory tests and standard treatment were provided according to the guidelines [37, 38]. The definition of stroke is a clinical syndrome of rapid onset, developing symptoms of focal or global cerebral dysfunction lasting ≥24 hours or leading to death with an apparent vascular cause [39]. Patients were excluded from the study when intracerebral hemorrhage was detected in the imaging scans. The exclusion criteria also involved the detection of malignancy or active autoimmune disease, infection symptoms, or temperature of the body >37.4°C. Patients who were unable to give consent to participate in the study due to any consciousness or speech impairment were also excluded from the study. The inflammatory metabolites and FFAs were assessed in all subjects. The liquid chromatography was used to perform the measurements of eicosanoids. Free fatty acids were detected with the use of gas chromatography (GC).

The group of patients consisted of 73 subjects, 33 of whom were males (45.2%). All subjects were Caucasians. The mean age was 60.7 years (min. 33, max. 83), 61 patients suffered from hypertension (83.6%), and 35 patients had diabetes or impaired fasting glucose tolerance (47.9%); coronary heart disease (CHD) was detected in 9 patients (12.3%) and BMI of 25 or more was detected in 58 subjects (79.4%), while smoking was present in 26 patients (33.1%). The treatment during the hospitalisation was as follows: 7 patients were on intravenous alteplase infusion, 14 were taking L-thyroxine (19.2%), 61 were taking hypotensives (83.6%), and 17 were taking hypoglycemics (23.3%). All patients received acetylsalicylic acid and statins.

The stroke etiology was classified according to the TOAST classification system, which describes stroke subtypes as follows: (1) large-artery atherosclerosis, (2) cardioembolism, (3) small vessel occlusion (lacunar), (4) other determined causes, and (5) undetermined cause [40].

### 2.2. Blood Collection, Free Fatty Acids, and Eicosanoid Detection

The collection of venous blood samples was obtained after seven days since the onset of the symptoms. The analyses of the eicosanoids and FFAs with the use of liquid and gas chromatography were performed after centrifugation, and the samples were stored at −80°C. The methyl esters of FFAs were isolated from serum with the use of the modified Folch and Szczuko methods [41, 42].

Detailed methodology of free fatty acid and inflammatory mediator detection was described elsewhere [43].

The plasma BDNF concentration was evaluated by using the R&D Systems ELISA kit (USA). The intra-assay coefficient of variation (CV) was <5%, and the detection limit was estimated at 20 pg/mL for the used kit.

### 2.3. Statistical Analysis

Statistical analyses were performed by means of the statistical software Statistica 13.1 (StatSoft, Cracow, Poland). The assumptions for the use of parametric or nonparametric tests were checked using the Shapiro-Wilk and Levene tests to evaluate the normality of the distributions and the homogeneity of variances, respectively. The significant differences in mean values between the groups were assessed by the one-way ANOVA. If the normality was violated, the Kruskal-Wallis nonparametric test was used. The correlation matrix was computed for combined BDNF and FFAs using Spearman's rank correlation (Spearman's rank correlation coefficient (*r*_s_)). Statistical significance was established at *p* < 0.05.

## 3. Results

**Figure 1:**
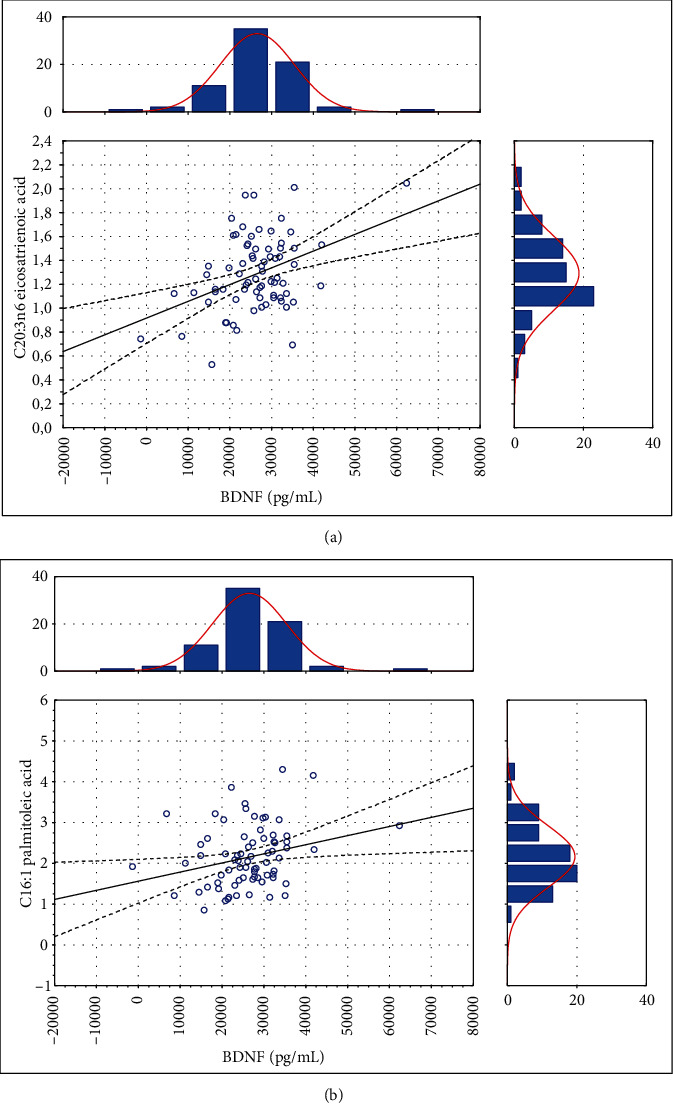
The relationship between (a) BDNF and C20:3n6 eicosatrienoic acid (*r*_s_ = 0.398, *p* < 0.05) and between (b) BDNF and C16:1 palmitoleic acid (*r*_s_ = 0.279, *p* < 0.05).

**Figure 2:**
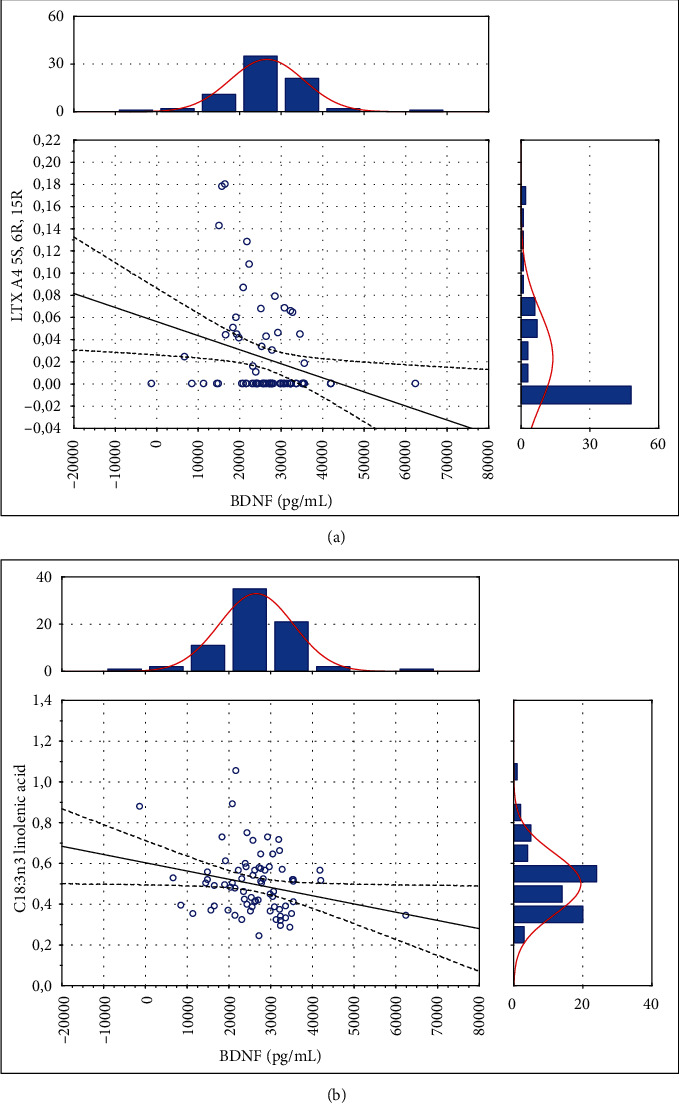
The relationship between (a) BDNF and lipoxin A4 15-epi-LxA4 A4 5S, 6R, 15R (*r*_s_ = −0.268, *p* < 0.05) and between (b) BDNF and C18:3n3 linolenic acid (*r*_s_ = −0.238, *p* < 0.05).

**Figure 3:**
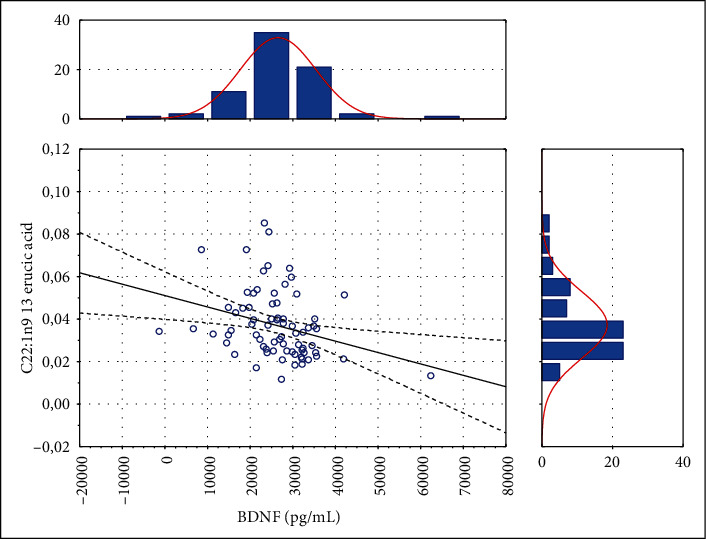
The relationship between BDNF and C22:1n9 13 erucic acid (*r*_s_ = −0.301, *p* < 0.05).

First, an analysis of the association between FFAs and serum BDNF was performed. The concentration of BDNF ranged from 6790 pg/mL to 62423 pg/mL (mean ± SD: 26317.55 pg/mL ± 8840.21 pg/mL) and was significantly related to certain FFA levels (Table 1). We detected a direct association regarding C16:1 palmitoleic acid and C20:3n6 eicosatrienoic acid, while a negative association was observed in C22:1n9 13 erucic acid and C18:3n3 linolenic acid. This means that BDNF release from the brain tissue can be modulated by changes in free fatty acids in stroke patients. The scatter plots of BDNF distribution of significant results of these associations are presented in Figures 1–3.

In the next step of the study, we analyzed the association between inflammatory metabolites of FFAs, i.e., eicosanoids, and BDNF in stroke patients. A negative association was detected between lipoxin A4 15-epi-LxA4 and BDNF level, which means that lipoxin level decreases with the increasing levels of BDNF. Other eicosanoids did not affect the BDNF levels (Table 2). A scatter plot of this significant association is presented in Figure 2.

Then, we performed the analysis to check any influence of the type of stroke according to the TOAST classification on BDNF level. The associations between BDNF and subtypes of stroke according to the TOAST classification are presented in Table 3. The number of patients in TOAST subgroups was as follows: large-artery atherosclerosis (*n* = 24), cardioembolism (*n* = 9), small vessel occlusion (lacunar) (*n* = 28), other determined causes (*n* = 0), and undetermined cause (*n* = 13). No impact of pathogenetical types of stroke on BDNF level was found.

*p* < 0.05: statistically significant differences. TOAST classification: 1—large-artery atherosclerosis, 2—cardioembolism, 3—small vessel occlusion (lacunar), and 5—undetermined cause.

## 4. Discussion

In our study, two fatty acids, i.e., dihomo-*γ*-linolenic acid (DGLA) and C16:1 palmitoleic acid, were found to be directly associated with the BDNF level. DGLA is an essential 20-carbon polyunsaturated fatty acid (PUFA) that is derived from linolenic acid. Arachidonic acid, DGLA, and eicosapentaenoic acid (EPA) are precursors of eicosanoids. Both AA and DGLA are substrates of the lipid-peroxidizing enzyme COX and form bioactive metabolites such as prostaglandin series 1 and 2 (PGs1 and PGs2). Prostaglandin 1 has anti-inflammatory properties, while prostaglandin 2 has proinflammatory ones. The major prostaglandin derived from DGLA is PGE_1_, and it can reduce vascular cell adhesion, inhibit vascular smooth muscle cell proliferation, and attenuate the development of atherosclerosis [44]. DGLA performs an anti-inflammatory function via the prostaglandins of series 1 and leukotrienes of series 3 formation and leukotrienes of series 4 inhibition and synthesis of specialized proresolving molecules [45]. Through its metabolites, DGLA also has antiplatelet, antithrombotic, and antiatherogenic functions [46]. DGLA attenuates atherosclerosis by the inhibition of the proatherogenic genes in macrophages, foam cell formation, and migration of monocytes. The antiatherogenic effects of DGLA are connected with the stimulation of cholesterol efflux from the foam cells and with the inhibition of LDL uptake by endocytosis and macropinocytosis. DGLA also exerts the effect on macrophages, improving their energetic mitochondrial profile due to the decrease in proton leak [47]. Despite the anti-inflammatory and antiatherogenic activity of DGLA, it was not proven to decrease the risk of stroke in the systematic review. It reduces the level of total cholesterol and has little or no effect on the reduction of the risk of heart attacks [48]. On the other hand, there is available data showing that in acute myocardial syndrome (ACS) patients, a higher DGLA level was found protective in the 7-year follow-up regarding myocardial infarct, stroke, or death [49]. The DGLA level is elevated in the animal model of brain ischemia, and DGLA-derived metabolites can reduce inflammation, although its role in the regulation of neurotransmission is not known [50]. In our study, DGLA was found to be correlated with the BDNF level, which may be connected with the common dietary and nutritional background. As mentioned in Introduction, the level of BDNF is influenced by FFAs and diet, while DGLA is increased in nonalcoholic fatty liver disease (NAFLD) in stroke patients [51]. We suggest that the association between DGLA and BDNF in our patients may result from both nutritional and inflammatory effects. Some consistency regarding the association with NAFLD refers to the C16:1 palmitoleic acid, the omega-7 monounsaturated fatty acid (MUFA), which was observed to be directly correlated with the BDNF level in our study. Palmitoleic acid is obtained from the diet or can be synthesized by the desaturation of palmitic acid (16:0). It circulates in the bloodstream within lipoproteins and acts as a lipokine modulating metabolic processes, such as enhancement of blood glucose disposal, attenuation of hepatic steatosis, improvement of the lipid profile, reduction of liver inflammation in NAFLD, inhibition of atherosclerosis as well as inflammatory cytokines, and expression of genes related to Toll-like receptor (TLR). The group of MUFA increases the level of HDL cholesterol and decreases triglycerides, but the effect on other lipids is inconsistent. MUFA reduces blood pressure and exerts a hypoglycemic effect in diabetic patients, but it affects neither the risk of CHD nor the risk of stroke [52, 53]. There is limited information in the available literature to link MUFA with BDNF. Interestingly, the results of human studies provided conflicting results compared to the animal studies. Most of the observations indicate no or negative correlations regarding metabolic and cardiovascular disorders. On the other hand, ambiguous but mostly beneficial effects of palmitoleic acid supplementation on the lipid profile were observed [54, 55]. The direct association between C16:1 palmitoleic acid and BDNF levels may be difficult to explain, but we suggest that the nutritional effect of both parameters is produced in an independent manner or there is a subsequent effect of C16:1 palmitoleic acid on BDNF. Such activation may be associated with the common pathogenetical background, together with DGLA, connected with NAFLD. These three parameters can be linked together by inflammation and FFA metabolism [56, 57]. Moreover, BDNF itself can directly impact the risk of cardiovascular disorders (CVD). It has already been shown that it affects the development and functioning of arterial baroreceptors, renin-angiotensin system, and endothelial nitric oxide synthase [24].

The C22:1n913 erucic acid is an omega-9 long-chain fatty acid, which may lead to lipidosis and heart failure due to poor mitochondrial beta-oxidation and accumulation, especially in the heart [58]. Consequently, the European Food Safety Authority established a daily intake limit for humans [59]. In single clinical studies, erucic acid was not associated with the incidence of stroke [60]. There is no available data regarding any link between BDNF and erucic acid. The negative correlation between erucic acid and BDNF in our study indicates that there is a potential association between these factors. Erucic acid is the ligand of the transcription factor peroxisome proliferator-activated receptor delta (PPAR-*δ*). The PPAR isotypes *β*/*δ* are the most abundant in the central nervous system and crucial for neuronal maturation. Their expression affects the BDNF signaling [61]. There is a common molecular signaling mechanism in which PPAR-*γ* regulates BDNF promotor activity [62]. Hippocampal PPAR-*δ* is involved in neurogenesis and modulates the level of BDNF [63]. The family of PPAR receptors is stimulated by fatty acids and eicosanoids and acts as transcription factors regulating gene expression. They also regulate transcription through protein-protein interactions with NF-*κ*B [64, 65]. The nuclear factor kappa-light-chain enhancer of activated B cells (NF-*κ*B) is engaged in neurogenesis, synaptic transmission, and plasticity, while BDNF activates NF-kappa B in the neurons at higher concentrations [66, 67]. We suggest that there may be a link between erucic acid, PPAR, and NF-*κ*B that can be responsible for the results we observed in our study. This area of research, however, needs further laboratory and clinical studies to verify our presumptions. The BDNF level may be under the influence of n-3 PUFA through the activation of cAMP response element-binding protein (CREB). This pathway involves CREB as the main transcription factor regulating cellular survival and growth, neuroinflammation, and neuroprotection. The molecular phosphorylation of CREB depends on the activation of kinase systems: phosphokinase C (PKC), Ca^2+^ calmodulin-dependent kinase IV (CaMK IV), phosphokinase A (PKA), and mitogen-activated protein kinase (MAPK). There can be some relationship between p38MAPK/CREB/BDNF/TrkB (tropomyosin receptor kinase B) pathway activation and neuronal plasticity, oxidative stress, and neuroinflammation. These factors may be accountable for the effect of FFAs on BDNF [16, 68]. A certain central nervous system disorder pathomechanism may be associated with the dysfunction of the BDNF/TrkB system. This refers to depression, Alzheimer's disease, and autism [3]. There are several pathomechanisms in experimental and animal studies that can help to explain the role of diet, nutritional status, and FFAs in the BDNF level. DHA specifically activates the free fatty acid receptor 1 (FFA1), also known as the G protein-coupled receptor 40 (GPR40), and modulates BDNF expression in primary cortical neurons mediated by the extracellular receptor kinase (ERK) and P38-mitogen-activated protein kinase (MAPK) pathways. This process leads to the memory potentiating effects of DHA and the induction of BDNF expression in the hippocampus of metabolic syndrome mice [69]. The high-fat diet deleteriously affects cognition and synaptic function by oxidative damage, which inhibits BDNF expression. Subsequently, the expression of BDNF downstream effectors in the hippocampus are also inhibited, i.e., CREB and synapsin I [70]. Free fatty acids can inhibit BDNF synthesis. Increased clearance of circulating BDNF that occurs in the liver can also decrease the level of BDNF [71]. The neuroplasticity markers—CaMKII and synapsin I—can be under the influence of BDNF. They are responsible for learning, long-term potentiation, synapse maturation and formation, release of neurotransmitters, and synaptic plasticity [72].

We found a significant association between BDNF and eicosanoids only in relation to 15-epi-lipoxin A_4_ (15-epi LXA_4_), which showed a reverse association. Lipoxins (LX) are proresolving lipid mediators derived from arachidonic acid by the action of 5- and 15-lipoxygenease (5-LOX and 15-LOX, respectively) [73]. The bioactive form 15-epi-lipoxin A_4_ (15-epi LXA_4_) promotes the resolution of inflammation by inhibiting superoxide generation and polymorphonuclear leukocyte transmigration [63]. Its role in ischemic stroke is connected with the decrease in reactive oxygen species and proinflammatory cytokine and chemokine production, blocking the adhesion to and transmigration across the endothelium, inducing apoptosis, increasing anti-inflammatory interleukins, and promoting resolution of inflammation [67]. It also inhibits TGF-*β*1-dependent collagen secretion and the differentiation of fibroblasts into myofibroblasts [73]. In the molecular docking model, it was shown that lipoxin A4 (LXA4) interacted with BDNF [65]. A possible interaction between these molecules was also presented by other authors in the type 2 diabetes animal model [66]. We demonstrated a negative correlation between BDNF and 15-epi-lipoxin A_4_. Alas, there is limited data in the available literature to justify the exact pathomechanism of this finding.

We observed a reverse association between the omega-3 polyunsaturated fatty acid C18:3 linolenic acid and the BDNF level. This essential fatty acid is supposed to have anti-inflammatory and neuroprotective properties and additionally to decrease the risk of stroke [74, 75]. It has been shown in animal models that ALA increases the level of BDNF in the brain through the activation of TrkB receptors and NF-*κ*B [76–78]. Although the consumption of ALA by healthy individuals increases the level of BDNF, the association between the level of ALA and BDNF, especially in stroke patients, has been scantily documented so far [9]. The reverse association between ALA and BDNF detected in our study may reflect diverse metabolic processes in humans compared to animal models or may result from the affected metabolism of BDNF during stroke. We encourage further research into the correlation between the level of omega-3 FA and the BDNF level in stroke patients.

We also evaluated the potential impact of the types of stroke according to the TOAST classification on BDNF level, and we did not detect any significant associations. There is limited information in the literature regarding a potential association between BDNF and type of stroke. Potential association between the pathogenetic background of strokes and BDNF level may be of importance as this was suggested by other authors. Lower BDNF level was connected with an increased risk of cardiovascular diseases, and this may indicate pathogenetic association between BDNF and atherosclerosis and endothelial dysfunction [79, 80]. In the animal model, it was detected that repetitive ischemic episodes mimicking the embolic strokes provoked an increased expression of BDNF mRNA in rats [81].

There are theories that BDNF level and metabolism are involved in the aging process, but it is not clear why BDNF levels decrease during the aging process. BDNF can have protective function against cardiovascular and metabolic disorders. It is suggested that the lower levels of BDNF in aging individuals make them more susceptible to suffering from cardiovascular disorders. Moreover, the aging process that is connected with the changes in energy regulation can also be modulated by the BDNF [19, 28].

## 5. Conclusions

Changes in blood BDNF levels and signaling may be a potential common factor for the metabolic syndrome and atherosclerosis playing a role in the development of cardiovascular disorders. There is a pathogenetical association between BDNF and CVD. It can be additionally impacted by nutritional factors such as FFAs and their inflammatory metabolites. FFAs and eicosanoids can interfere with the BDNF level and the risk of CVD by modulating the BDNF metabolism and by affecting the neuroinflammatory status. Our understanding of the role of FFAs and their inflammatory metabolites in BDNF metabolism can add to the search for novel therapies in cardiovascular disorders such as stroke [19].

## Figures and Tables

**Table 1:** Correlations between BDNF and FFA levels.

FFA	Correlation matrix	FFA	Correlation matrix
C13:0 tridecanoic acid	-0.057	C20:0 arachidic acid	0.052
C14:0 myristic acid	-0.043	C22:1/C20:1 Cis11-eicosanic acid	0.145
C14:1 myristolenic acid	-0.021	C20:2 Cis-11-eicodienoic acid	0.123
C15:0 pentadecanoic acid	-0.006	C20:3n6 eicosatrienoic acid	0.398^∗^
C15:1 cis-10-pentadecanoic acid	-0.095	C20:4n6 arachidonic acid	0.074
C16:0 palmitic acid	-0.042	C20:3n3 Cis-11-eicosatrienoic acid	-0.084
C16:1 palmitoleic acid	0.264^∗^	C20:5n3 eicosapentaenoic acid	0.057
C17:0 heptadecanoic acid	-0.032	C22:0 behenic acid	-0.095
C17:1 cis-10-heptadecanoid acid	-0.191	C22:1n9 13 erucic acid	-0.301^∗^
C18:0 stearic acid	-0.136	C22:2 cis-docosadienoic acid	0.225
C18:1n9 ct oleic acid	0.114	C23:0 tricosanoic acid	-0.17
C18:1 vaccenic acid	0.229	C22:4n6 docosatetraenoate	-0.049
C18:2n6c linoleic acid	-0.119	C22:5w3 docosapentaenate	0.134
C18:2n6t linoleic acid	-0.096	C24:0 lignoceric acid	-0.131
C18:3n6 gamma linoleic acid	0.128	C22:6n3 docosahexaenoic acid	-0.151
C18:3n3 linolenic acid	-0.238^∗^	C24:1 nervonic acid	-0.02
C18:4 stearidonate	-0.003		

^∗^ *p* < 0.05: statistically significant correlation matrix (Spearman's rank correlation).

**Table 2:** Correlation matrix between BDNF and eicosanoid levels.

Eicosanoids	Correlation matrix
Resolvin E1	0.039
Prostaglandin E2	-0.142
Resolvin D1	-0.013
Lipoxin A4 LxA4 5S, 6R	-0.026
Lipoxin A4 15-epi-LxA4 A4 5S, 6R, 15R	-0.268^∗^
Protectin D1	0.113
Maresin 1	-0.118
Leukotriene B4	-0.041
18RS HEPE	0.084
16RS HETE	0.189
13S HODE	-0.022
9S HODE	-0.015
15S HETE	-0.129
17RS HDHA	0.008
12S HETE	-0.04
5-Oxo-ETE	-0.045
5 HETE	0.014

^∗^ *p* < 0.05: statistically significant correlation matrix (Spearman's rank correlation).

**Table 3:** The comparison of subgroups (*n* = 73) in regard to the TOAST classification and BDNF level (mean ± SD).

Subgroups in the TOAST classification	BDNF (pg/mL)	*p* value
1 vs. 2	26244 ± 7706 vs. 26774 ± 5595	0.852
1 vs. 3	26244 ± 7706 vs. 26979 ± 6924	0.721
1 vs. 5	26244 ± 7706 vs. 26051 ± 13251	0.955
2 vs. 3	26774 ± 5595 vs. 26979 ± 6924	0.936
2 vs. 5	26774 ± 5595 vs. 26051 ± 13251	0.879
3 vs. 5	26979 ± 6924 vs. 26051 ± 13251	0.771

## Data Availability

Data are available on request.
